# The utility of measles and rubella IgM serology in an elimination setting, Ontario, Canada, 2009–2014

**DOI:** 10.1371/journal.pone.0181172

**Published:** 2017-08-29

**Authors:** Shelly Bolotin, Gillian Lim, Vica Dang, Natasha Crowcroft, Jonathan Gubbay, Tony Mazzulli, Richard Schabas

**Affiliations:** 1 Public Health Ontario, Toronto, Ontario, Canada; 2 Dalla Lana School of Public Health, University of Toronto, Toronto, Ontario, Canada; 3 Department of Laboratory Medicine and Pathobiology, Faculty of Medicine, University of Toronto, Toronto, Ontario, Canada; 4 Department of Pediatrics, Faculty of Medicine, University of Toronto, Toronto, Ontario, Canada; 5 Division of Infectious Diseases, The Hospital for Sick Children, Toronto, Ontario, Canada; 6 Division of Infectious Diseases, Department of Medicine, Faculty of Medicine, University of Toronto, Toronto, Ontario, Canada; 7 Mount Sinai Hospital, Toronto, Ontario, Canada; 8 Hastings Prince Edward Public Health, Belleville, Ontario, Canada; RIVM, NETHERLANDS

## Abstract

In Canada, measles was eliminated in 1998 and rubella in 2000. Effective measles and rubella surveillance is vital in elimination settings, hinging on reliable laboratory methods. However, low-prevalence settings affect the predictive value of laboratory tests. We conducted an analysis to determine the performance of measles and rubella IgM testing in a jurisdiction where both infections are eliminated.

21,299 test results were extracted from the Public Health Ontario Laboratories database and 1,239 reports were extracted from the Ontario Integrated Public Health Information System (iPHIS) from 2008 and 2010 for measles and rubella, respectively, to 2014. Deterministic linkage resulted in 658 linked measles records (2009–2014) and 189 linked rubella records (2010–2014). Sixty-six iPHIS measles entries were classified as confirmed cases, of which 53 linked to laboratory data. Five iPHIS rubella entries were classified as confirmed, all linked to IgM results. The positive predictive value was 17.4% for measles and 3.6% for rubella. Sensitivity was 79.2% for measles and 100.0% for rubella. Specificity was 65.7% for measles and 25.8% for rubella.

Our study confirms that a positive IgM alone does not confirm a measles case in elimination settings. This has important implications for countries that are working towards measles and rubella elimination.

## Introduction

Measles has been eliminated in Canada since 1998 [1,2], with sporadic cases and outbreaks linked to importations of the virus [3]. Since then, only a handful of small outbreaks have occurred in Ontario [1,4], supporting sustained elimination in the province. Rubella has been eliminated since 2000 in Canada [1,5], and verified as eliminated in the whole region of The Americas in 2015 [6], with only sporadic single imported cases in Ontario [1].

In Ontario, a single dose of the combined measles-mumps-rubella (MMR) vaccine was implemented in 1975, followed by a two-dose program in 1996 [7]. The first dose of the MMR vaccine has routinely been administered at 12 months of age. The timing of the second dose has varied between 18 months and 4–6 years of age [8], and is currently given at 4–6 years in a combined vaccine that also delivers a second dose of varicella antigen [9]. Coverage data are somewhat limited in the absence of a vaccine registry, but vaccination coverage figures indicate that >88% of 7 year olds in Ontario in 2012/13 had received two doses of measles containing vaccine and at least one dose of rubella containing vaccine [9].

Surveillance of measles and rubella is based on a combination of clinical symptoms and laboratory diagnosis [10,11], and is used to guide public health policies and interventions. Effective surveillance is extremely important in elimination settings, where even a single case is considered a public health priority. Successful surveillance efforts hinge on reliable laboratory methods that are adapted to the setting.

In high incidence settings, measles and rubella diagnoses can be made based on clinical data [12,13]. However, in low incidence and elimination settings, diagnosis of measles and rubella infections requires confirmation using laboratory testing. Virus isolation is possible from several specimen types, but is technically demanding and can be unreliable [13,14], and currently in many settings is mainly used for surveillance of circulating genotypes [13]. For measles, direct immunofluorescence methods from nasopharyngeal or urine specimens have also been used, but since the specimen must be collected soon after rash onset they have limited utility [12,13]. Polymerase chain reaction (PCR) can be used for direct detection of virus, but may not be available in all settings [13]. Avidity testing and plaque reduction neutralization testing of high-avidity samples may provide important additional information and also be a means of diagnosis in low-burden settings [15,16]. Serological Immunoglobulin M (IgM) testing using either capture or indirect enzyme immunoassays [14] has been a standard laboratory method for diagnosis of acute measles and rubella infections, since it is more readily available than PCR, and faster and less technically challenging than viral culture [17]. In addition to IgM testing using sera specimens, dried blood spot specimens and oral fluid samples are also used in some jurisdictions [18]. The majority of measles cases are IgM positive in the first two days after the onset of rash, with 90% of cases positive three to five days after the rash appears [14]. For rubella, IgM antibodies appear within 3–4 days after rash onset and are sometimes detectable up to two months after illness. The timing of specimen collection is of utmost importance, because specimens may test negative if collected too early after rash onset. If collected within the first three days of rash, up to 30% of measles IgM tests and 50% of rubella IgM tests may be false-negative, respectively [14]. For rubella in particular, an additional challenge is that 20–50% of cases are asymptomatic, meaning that testing would only be performed if there is an epidemiological link [19]. Several commercial assays are available to measure measles and rubella IgM. These are extensively used by clinical and public health laboratories, and are the primary confirmatory test used by the Pan American Health Organization for surveillance of measles elimination in the Americas, as well as the World Health Organization (WHO) Global Measles Laboratory network [14].

The low incidence of measles and rubella in elimination settings presents a challenge for diagnostic serological IgM testing, because the positive predictive value (PPV) of a test depends on the prevalence of disease [20]. Few jurisdictions have access to comprehensive laboratory and public health data that can be used to estimate the probability of false positive results. This study was undertaken at the request one of the authors, who is a local medical officer of health, who raised concerns following involvement in two consecutive measles investigations triggered by false-positive IgM test results. His initial Bayesian analysis concurred with the well-known phenomenon that even with a test of very high specificity such as measles or rubella IgM [21], when the prevalence of disease is low the positive predictive value of any test is too low to confirm a diagnosis in the absence of strong supporting evidence (such as a travel history) [12,22]. We conducted an analysis to determine the PPV of measles and rubella IgM results in the context of a jurisdiction such as Ontario, Canada, where both infections are eliminated.

## Methods

### Laboratory testing and epidemiological data

In Ontario, the Public Health Ontario (PHO) Laboratories perform all IgM serological testing for both measles and rubella. Since October 2009, the PHO Laboratories has been using measles and rubella IgM immunoassays manufactured by Euroimmun AG, Luebeck, Germany. Prior to this, Dade Behring Enzygnost anti-measles virus and anti-rubella virus IgM immunoassays (Dade Behring, Marburg, Germany) were used. Evaluation of the Euroimmun platform prior to implementation showed that it performed similarly to the Enzygnost assay. Results for specimens that were submitted for measles and rubella testing were extracted from the PHO Laboratory Information System. Prenatal tests, PCR or viral culture tests, IgG results and specimens that were also vaccine-strain PCR positive (as tested at the National Microbiology Laboratory in Winnipeg, Manitoba, Canada) were excluded. Measles and rubella IgM serology results were available as of December 2008 and March 2010, respectively; the latest result available for analysis was November 2014. Test-level results were summarised into patient-level results using a combination of patient name and date of birth. Multiple test results per patient were collapsed using the following hierarchy: reactive > non-reactive > indeterminate. Multiple test results were considered as belonging to the same episode if they occurred within four months.

In Ontario, reporting of measles and rubella cases by laboratories and/or physicians to public health authorities is mandatory [23]. Entries from all suspected reports of measles from December 2008 –November 2014 and rubella from March 2010 –November 2014 were extracted from Ontario’s Integrated Public Health Information System (iPHIS), which is managed by PHO. These included entries with any case-definition classification (‘confirmed’, ‘probable’, ‘suspect, ‘does not meet’, ‘person under investigation’), as well as symptom, immunisation and severity variables. Duplicate or erroneous reports were excluded. We removed measles cases from December 2008 from the analysis as they were associated with an outbreak and not representative of the usual burden of disease in Ontario.

### Data linkage and analysis

We conducted deterministic linkage of iPHIS entries with patient-level serology results by first collapsing test-level laboratory results into patient-level results, then linking patient-level laboratory results with iPHIS entries on the basis of name and date of birth. Positive and negative predictive values as well as sensitivity and specificity of a reactive serology result were determined by using a confirmed case of measles or rubella as reported in iPHIS as the gold standard for disease. We chose confirmed cases reported in iPHIS as the gold-standard because all cases entered in the system are subjected to rigorous case investigations that account for clinical, epidemiological and laboratory factors. Cases must meet a stringent case definition to be entered as ‘confirmed’, such as positive laboratory confirmation including virus isolation, PCR, paired IgG serology, or IgM serology with an epidemiological link to a confirmed case or appropriate travel history, or alternatively, clinical symptoms and an epidemiological link to a laboratory confirmed case [10,11]. All case definitions other than ‘confirmed’ were excluded in the analysis. All data manipulation and analyses were conducted in SAS version 9.3, apart from Chi-square testing, which was performed using the Quantpsy online tool [24]. Both data linkage steps as well as subsequent calculations were independently replicated by two analysts to ensure their accuracy.

### Ethics approval

This study received approval from the Public Health Ontario Research Ethics Board. It used already existing diagnostic laboratory testing results from the PHO Laboratories as well as public health information in iPHIS, and therefore participants did not provide written or verbal informed consent to be included. The research relied exclusively on the secondary use of information rendered non-identifiable for all practical purposes. Consent was not required as the conditions of Canada’s Tri-Council Policy Statement: Ethical Conduct for Research Involving Humans 2, Article 5.5B were satisfied.

## Results

**Fig 1:**
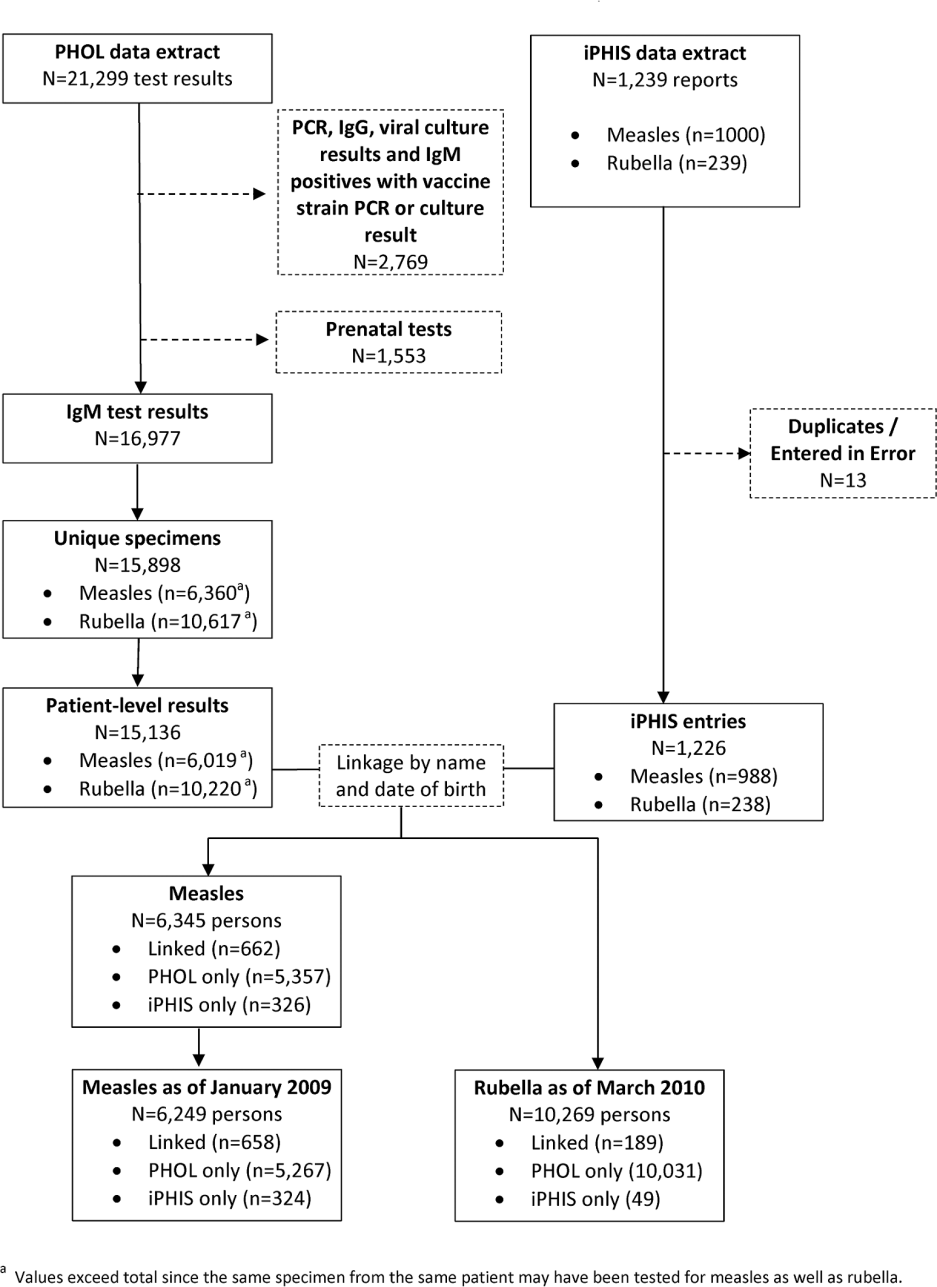
Data extraction, linkage and analysis flowchart.

A total of 21,299 measles and rubella laboratory test results were extracted from the PHO laboratory information system (Fig 1). After removal of PCR tests, IgG tests, viral culture tests, positive IgM results that also had vaccine-strain positive viral culture and PCR results (n = 2,769) as well as prenatal results (n = 1,553), 16,977 IgM results remained, belonging to 15,136 individuals. Of these, 6,019 were tested for measles and 10,220 were tested for rubella. Some individuals were tested for both measles and rubella.

A total of 1,239 entries were extracted from iPHIS, each representing a patient investigated for measles or rubella by public health authorities. After removal of duplicates and erroneous entries (n = 13), 1,226 entries remained. Of these, 988 were measles-related and 238 were rubella-related.

Laboratory results were linked to iPHIS entries by name and date of birth. For measles, we were able to link a total of 662 entries. These comprised 67.0% (662/988) of iPHIS measles entries and 11.0% (662/6019) of PHOL measles entries. For rubella, we were able to link a total of 189 entries. These comprised 79.4% (189/238) of all iPHIS rubella entries and 1.8% (189/10,220) of laboratory rubella results. Linked measles results from December 2008 were associated with an outbreak and are not representative of the usual setting in Ontario [7]. We therefore removed them from the analysis, making January 2009 the start of the analysis period.

Linked measles records as of January 2009 (n = 658) had a median age of 5.6 years, older than iPHIS-only entries (median age 5.4 years) and younger than laboratory-only results (median age 28.1 years, p<0.0001) (Table 1). Linked rubella entries had a median age of 27.1 years (Table 2)—older than iPHIS-only entries and younger than laboratory-only results, which had median ages of 8.8 and 29.5 years, respectively (p = 0.0001). For measles, roughly half of the linked records and iPHIS-only entries were females, while 60.4% (3179/5267) of laboratory patient-level results were female (p<0.0001). The majority of rubella records, ranging from 73.5–80.3%, were female regardless of data source or linkage status (p = 0.008). More linked measles records reported fever and rash than iPHIS-only cases (p<0.0001), and less were immunised (p = 0.02) (no symptom or immunisation information is available from laboratory sources). Fever was reported in slightly more linked rubella records than entries that were found only in iPHIS (p = 0.85). Patients with linked records reported less rash (p = 0.49) and rubella immunisation (p = 0.11) than those with entries in iPHIS only.

**Table 1:** Profile of measles cases by data source, Ontario, Canada, 2009–2014.

	Person-level entries by data Source (N = 6,345)			
	Linked data n = 658	iPHIS only n = 324	PHOL only n = 5,267	p-value
**Age (years)**				
**Median**	5.6	5.4	28.1	<0.0001 ^a^
**Range** **0–1** **1–9** **10–19** **20–39** **40–59** **60+** **unknown**	0.2–86.7 87 318 86 99 49 19 0	0.03–73.4 32 200 43 32 14 3 0	0.03–96.4 151 944 646 2098 1060 356 12	
**Sex N (%)**				
**Female**	317 (48.2)	160 (49.4)	3,179 (60.4)	<0.0001 ^b^
**Male**	339 (51.5)	161 (49.7)	1,926 (36.6)	
**Unknown**	2 (0.3)	3 (0.9)	162 (3.1)	
**Any reported fever N (%)**				
**Yes**	361 (54.9)	112 (34.6)	n/a	<0.0001 ^b^
**No**	297 (45.1)	212 (65.4)	n/a	
**Any reported rash N (%)**				
**Yes**	420 (63.8)	134 (41.4)	n/a	<0.0001 ^b^
**No**	238 (36.2)	190 (58.6)	n/a	
**Any measles immunization N (%)**				
**Yes**	160 (24.3)	102 (31.5)	n/a	0.02 ^b^
**No**	498 (75.7)	222 (68.5)	n/a	

^a^—Wilcoxon-Mann-Whitney test

^b^—Chi-square test

**Table 2:** Profile of rubella cases by data source, Ontario, Canada, 2009–2014.

	Person-level entry by data Source (N = 10,269)				
	Linked data n = 189	iPHIS only n = 49		PHOL only n = 10,031	p-value
**Age (years)**					
**Median** **Range** **0–1** **1–9** **10–19** **20–39** **40–59** **60+** **Unknown**	27.1 0.2–83.7 11 39 18 85 28 7 1	8.8 0.03–66.9 6 19 3 17 2 1 1		29.5 0–100 1086 502 712 6449 1048 206 28	0.0001 ^a^
**Sex N (%)**					
**Female**	149 (78.8)	36 (73.5)		8,051 (80.3)	0.008 ^b^
**Male**	40 (21.2)	13 (26.5)		1645 (16.4)	
**Unknown**	0 (0)	0 (0)		335 (3.3)	
**Any reported fever N (%)**					
**Yes**	41 (21.7)	10 (20.4)		n/a	0.85 ^b^
**No**	148 (78.3)	39 (79.6)		n/a	
**Any reported rash N (%)**					
**Yes**	56 (29.6)	17 (34.7)		n/a	0.49 ^b^
**No**	133 (70.4)	32 (65.3)		n/a	
**Any rubella immunization N (%)**					
**Yes**	25 (13.2)	11 (22.4)		n/a	0.11 ^b^
**No**	164 (86.8)	38 (77.6)		n/a	

^a^—Wilcoxon-Mann-Whitney test

^b^—Chi-square test

**Fig 2:**
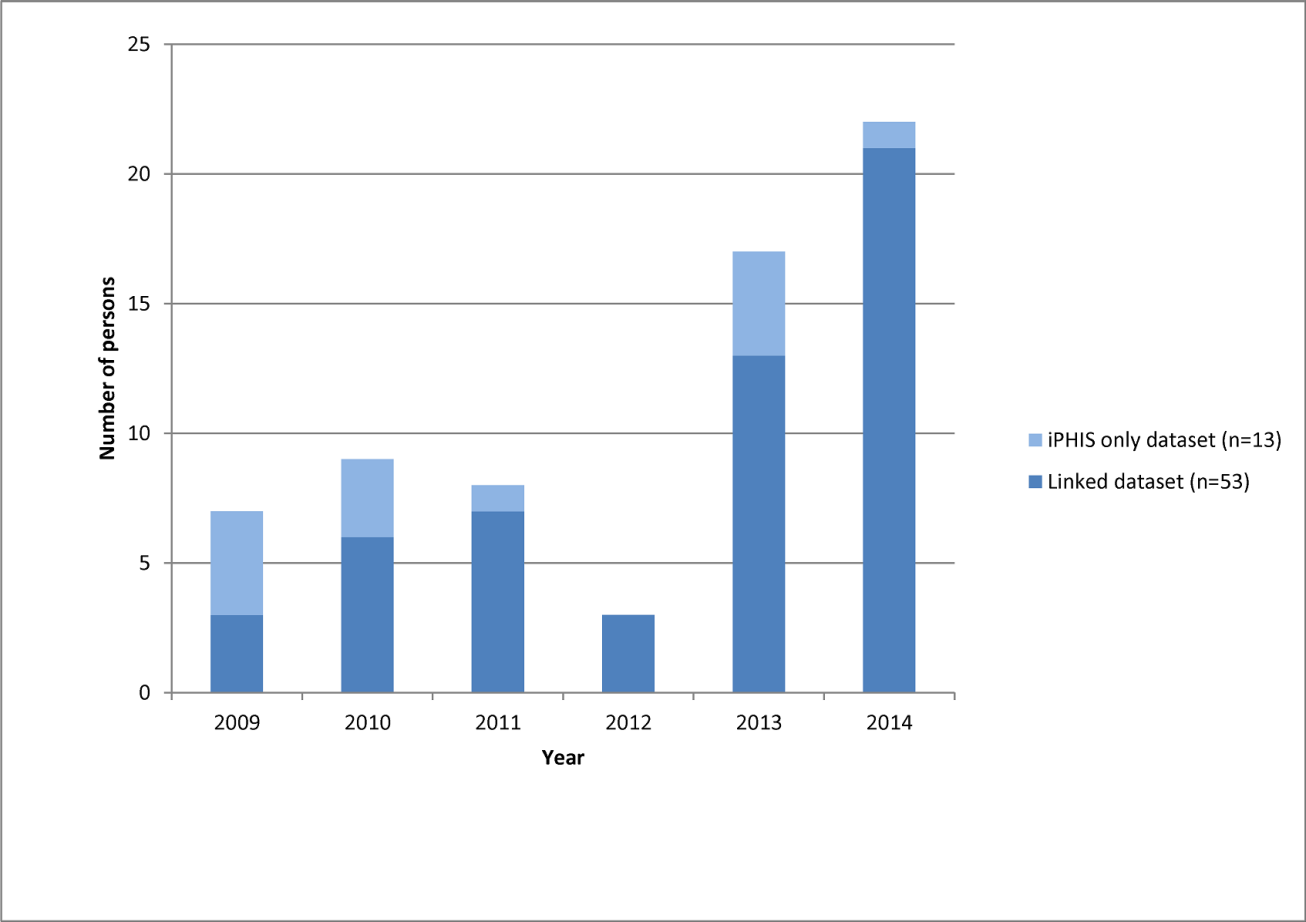
Distribution of confirmed measles cases in iPHIS by year and linkage status, Ontario, Canada 2009–2014, (N = 66).

The number and proportion of measles IgM reactive results varied by year depending on the occurrence of known local outbreaks. Linked records had a higher proportion of reactive IgM results, with 243/658 (36.9%) of the linked records having a reactive IgM compared to only 76/5267 (1.4%) of laboratory-only results (p<0.0001). A total of 66 measles entries classified as ‘confirmed’ were entered in iPHIS from 2009 to 2014. Of these, 53/66 (80.3%) were linked to laboratory results, of which 42 were IgM reactive. The remaining 13 iPHIS-only entries (19.7%) between were likely symptomatic cases that were epidemiologically linked, or diagnosed by viral culture only (Fig 2).

We saw a similar pattern for rubella. The number and proportion of IgM reactive results differed from year to year, although there was less variation than for measles. Linked records had a higher proportion of reactive IgM results, with 140/189 (74.1%) of the linked entries having a reactive IgM compared to only 142/10,031 (1.4%) laboratory-only results (p<0.0001). There were only five entries classified as ‘confirmed’ cases in iPHIS between 2010 and 2014 (one per year), all of which were linked to IgM reactive laboratory results.

The PPV of a reactive IgM for measles during the study period was 17.4% (Table 3). This increased to 28.0% if fever was reported and 26.5% with rash. The measles IgM test sensitivity was 79.2% overall, and did not change significantly in those who reported fever (78.4%) or rash (78.8%). Measles IgM test specificity was 65.7%, and was similar in those who reported fever (65.2%) or rash (67.5%).

**Table 3:** Performance of measles IgM serology test using linked dataset, Ontario, Canada, 2009–2014.

IgM serology result	iPHIS Classification		Positive predictive value (%)	Negative predictive value (%)	Sensitivity (%)	Specificity (%)
	Confirmed	Not-confirmed^a^				
**Positive**	42	199	17.4	97.2	79.2	65.7
**Negative**	11	381				
**By age (years)**						
**0–1**			20.6	100.0	100.0	63.5
**1–19**			12.8	97.9	79.2	64.2
**20–39**			26.5	92.1	64.3	69.9
**40+**			29.2	97.7	87.5	71.7
**Any reported fever**						
**Yes**			28.0	94.6	78.4	65.2
**No**			2.0	100.0	100.0	66.2
**Any reported rash**						
**Yes**			26.5	95.6	78.8	67.5
**No**			1.2	100.0	100.0	62.9
**Any measles immunization**						
**Yes**			11.1	95.9	60.0	66.0
**No**			19.3	97.6	83.7	65.6

^a^- includes ‘Does not meet’, ‘suspect’ and ‘Person under investigation’ classifications, excludes ‘probable’ classification

For rubella, the PPV of a reactive IgM test was 3.6%, increasing to 15.0% with reported fever and 15.4% with reported rash (Table 4). The test sensitivity was 100%, however, the specificity was 25.8%, increasing to 54.1% and 57.7% in cases with reported fever and rash, respectively.

**Table 4:** Performance of rubella IgM serology test using linked dataset, Ontario, Canada, 2009–2014.

IgM serology result	iPHIS Classification		Positive predictive value (%)	Negative predictive value (%)	Sensitivity (%)	Specificity (%)
	Confirmed	Not-confirmed^a^				
**Positive**	5	135	3.6	100.0	100.0	25.8
**Negative**	0	47				
**By age (years)**						
**0–1**			0.0	100.0	-	40.0
**1–19**			4.3	100.0	100.0	60.0
**20–39**			3.9	100.0	100.0	9.8
**40+**			3.0	100.0	100.0	5.9
**Any reported fever**						
**Yes**			15.0	100.0	100.0	54.1
**No**			1.7	100.0	100.0	18.6
**Any reported rash**						
**Yes**			15.4	100.0	100.0	57.7
**No**			0.9	100.0	100.0	13.1
**Any rubella immunization**						
**Yes**			7.1	100.0	100.0	45.8
**No**			3.2	100.0	100.0	22.8

^a^- includes ‘Does not meet’, ‘suspect’ and ‘Person under investigation’ classifications, excludes ‘probable’ classification

## Discussion

Measles and rubella IgM serology is key for both clinical diagnosis and for surveillance purposes, and is a recommended diagnostic by the WHO [14]. However, it is axiomatic that even a highly specific and sensitive test has a poor PPV when the prevalence of disease is low, as is the case in countries where diseases such as measles and rubella have been eliminated [25]. Only one region in the world has thus far succeeded in eliminating measles and rubella, the Americas, and within that region only some jurisdictions have access to both comprehensive laboratory data and robust public health data to be able to estimate the PPV. In Ontario, all measles and rubella IgM testing is performed at PHO Laboratories and public health investigations are recorded in the provincial case-management tool iPHIS, enabling access to this unique dataset.

Our analyses of laboratory and public health investigation data, using thoroughly investigated iPHIS data as the gold-standard, showed that the PPV for both measles and rubella IgM testing is low in Ontario. Although this would be expected in a region with a low burden of measles and rubella disease, the degree to which we observed this was striking. The PPV was lower for rubella than for measles, at only 3.6%. This is likely because there were only five confirmed cases reported in iPHIS from 2010–2014, but 10,220 laboratory tests performed. With such high volumes of testing and such low disease prevalence, even a laboratory test with superb performance will falter. Interestingly, the PPV did not increase substantially when clinical symptoms such as fever or rash were taken into consideration, perhaps due to the non-specific nature of these symptoms.

Like the PPV, the test sensitivities for both measles and rubella were also mediocre but higher than the specificities, since in jurisdictions with very little disease there is also little opportunity for false negatives. Once again, this was particularly apparent with rubella, which had a test specificity of 25.8%, suggesting that three-quarters of all specimens that tested positive for IgM tests are false-positives.

Positive IgM tests for measles or rubella represent one of four scenarios [26]. The first is true infection, which is rare in low-burden settings. The second is as a result of recent MMR vaccination, which results in rash in approximately 5% of vaccinees [27]. The third is due to a false positive result in someone who was tested while healthy (for example occupational screening), in which the IgM was incorrectly requested. The fourth, in someone who is symptomatic, is due to another exanthemous disease [28–31]. As demonstrated in this study, combining laboratory test results with clinical symptoms does not always elucidate a diagnosis, as fever and rash are non-specific symptoms, and measles can present mildly in previously vaccinated cases [27]. Epidemiological information such as previous infection or recent exposure can sometimes be helpful for diagnosis if a known epidemiological link exists. However, since measles is incredibly infectious [32], the absence of an epidemiological link does not rule out infection, even in elimination settings [26]. This means that, even in low burden settings, it is important that positive IgM results are thoroughly and rapidly investigated to determine which of these scenarios they represent, before concluding that they represent false-positive tests. Additional laboratory testing is integral for providing additional information for resolving these cases [22,29]. RT-PCR can be used to detect measles in urine specimens up to several weeks after disease onset, providing a non-invasive specimen option, which can also be used for genotyping [33]. Avidity testing can provide important contextual information, since samples from recently infected individuals would have low IgG antibody avidity, while those from those infected or immunized in the past would have high IgG antibody avidity [16]. For measles, plaque reduction neutralization tests of high avidity sera can be used diagnostically, with high concentrations of neutralizing antibodies being indicative of true cases [15]. IgG testing as well as IgM testing for other viruses which may have cross-reactivity to measles or rubella IgM test would also provide important information [22]. Ruling out false positive results due to assay interference, where a component of the patient’s serum (for example, rheumatoid factor) interacts with a component of the assay, should also be done by sample dilution or chemical or heat treatment [22].

Others have described the occurrence of false-positive measles and rubella IgM test results, including a case report of a patient who was diagnosed with parvovirus infection after initially testing positive for measles IgM [34], as well as reports of false-positive IgM results that were resolved with additional IgG testing [35,36]. However, to our knowledge ours is the first study that used confirmed cases of measles and rubella investigated by public health authorities as the gold standard to calculate IgM test performance.

There are several limitations to this study. Some characteristics of our setting may limit generalisability, particularly the high numbers of measles and rubella IgM tests are being performed in an elimination setting. We do not have further details on why testing was performed beyond being aware that large numbers of students and healthcare workers are screened and that some physicians may also test patients post-immunisation, despite the fact that IgM serology should never be used to screen otherwise healthy individuals. Physicians sometimes order measles and rubella IgM for any patient with rash. Cross reactivity and false positives with measles, rubella and other viral IgM tests also result in additional repeat testing. Since we excluded other laboratory tests from our analysis, such as IgG serology and PCR results for measles, rubella and other differential tests, and instead used confirmed iPHIS cases as the gold-standard, we are unable to directly validate our reported IgM sensitivity and specificity against other laboratory diagnostic methods. However, since the ‘confirmed’ case definition in iPHIS must meet strict criteria including other laboratory tests performed, we do not think that this affected our results significantly. Our findings underscore the need for technological solutions to improve the information provided by clinicians on their reasons for testing to help laboratories perform the appropriate tests and provide correct interpretation. Of the IgM test results, 319 were measles IgM positive and 282 were rubella IgM positive. However, there were only 53 linked, confirmed cases of measles (of which 42 were IgM reactive) and five linked, confirmed cases of rubella during the study period. It is very unlikely that the IgM positive patients represent true cases. Without further information such as the reason for testing, other laboratory tests performed (i.e.–PCR apart from vaccine-strain positive or IgM testing for other viruses) epidemiological or clinical data we do not know why they tested positive or why they were not reported to public health authorities. The fact that they were not reported supports testing being conducted as part of routine screening of healthy individuals, which should not be occurring but has been described by others [25]. Alternatively, it is possible that in the context of available clinical or epidemiological data the microbiologists performing the test recognized the result as a false positive, performed additional testing if required, and decided not to report the result as a confirmed case.

The finding that the PPV for measles and rubella IgM is so low is of global importance for other countries and regions that are working towards elimination. Although the concept is familiar [25,35], few studies have quantified this issue. The low PPVs have implications for individual clinical diagnosis as well as surveillance case definitions. In Ontario, the measles case definition was modified in 2014 so that a positive IgM does not confirm a case unless accompanied by an epidemiological link to another case, an appropriate travel history or virus detection by culture or PCR [10]. A positive IgM alone is also not accepted as confirmatory testing for rubella [11]. Our findings indicate that Ontario’s case definition is correct in not accepting a positive IgM result for a case to be confirmed but requiring additional information such as an epidemiologic link. Outside of Canada, other countries and regions should also plan to change their case definition once they have achieved elimination to avoid a high burden of false positive cases due to low PPV of IgM in a low prevalence setting.
